# Histogram analysis of prostate cancer on dynamic contrast-enhanced magnetic resonance imaging: A preliminary study emphasizing on zonal difference

**DOI:** 10.1371/journal.pone.0212092

**Published:** 2019-02-12

**Authors:** Chih-Ching Lai, Pin-Hsun Huang, Fu-Nien Wang, Shu-Huei Shen, Hsin-Kai Wang, Hsian-Tzu Liu, Hsiao-Jen Chung, Tzu-Ping Lin, Yen-Hwa Chang, Chin-Chen Pan, Shin-Lei Peng

**Affiliations:** 1 Department of Biomedical Engineering and Environmental Sciences, National Tsing Hua University, Hsinchu, Taiwan; 2 Department of Radiology, Taipei Veterans General Hospital, Taipei, Taiwan; 3 School of Medicine, Taipei, National Yang-Ming University, Taipei, Taiwan; 4 Department of Urology, Taipei Veterans General Hospital, Taipei, Taiwan; 5 Department of Pathology, Taipei Veterans General Hospital, Taipei, Taiwan; 6 Department of Biomedical Imaging and Radiological Science, China Medical University, Taichung, Taiwan; National Health Research Institutes, TAIWAN

## Abstract

**Background:**

This study evaluated the performance of histogram analysis in the time course of dynamic contrast-enhanced magnetic resonance imaging (DCE-MRI) for differentiating cancerous tissues from benign tissues in the prostate.

**Methods:**

We retrospectively analyzed the histograms of DCE-MRI of 30 patients. Histograms within regions of interest(ROI) in the peripheral zone (PZ) and transitional zone (TZ) were separately analyzed. The maximum difference wash-in slope (MWS) and delay phase slope (DPS) were defined for each voxel. Differences in histogram parameters, namely the mean, standard deviation (SD), the coefficient of variation (CV), kurtosis, skewness, interquartile range (IQR), percentile (P10, P25, P75, P90, and P90P10), Range, and modified full width at half-maximum (mFWHM) between cancerous and benign tissues were assessed.

**Results:**

In the TZ, CV for ROIs of 7.5 and 10mm was the only significantly different parameter of the MWS (*P* = 0.034 and *P* = 0.004, respectively), whereas many parameters of the DPS (mean, skewness, P10, P25, P50, P75 and P90) differed significantly (*P* = <0.001–0.016 and area under the curve [AUC] = 0.73–0.822). In the PZ, all parameters of the MWS exhibited significant differences, except kurtosis and skewness in the ROI of 7.5mm(*P* = <0.001–0.017 and AUC = 0.865–0.898). SD, IQR, mFWHM, P90P10 and Range were also significant differences in the DPS (*P* = 0.001–0.035).

**Conclusion:**

The histogram analysis of DCE-MRI is a potentially useful approach for differentiating prostate cancer from normal tissues. Different histogram parameters of the MWS and DPS should be applied in the TZ and PZ.

## Introduction

Prostate cancer is the most common malignancy in men, accounting for 26% of newly diagnosed cancer cases and 9% of cancer-related deaths in the United States in 2015 [1]. In recent years, multiparametric magnetic resonance (MR) imaging (mp-MRI), which comprises high-resolution T2-weighted imaging(T_2_WI), diffusion-weighted imaging (DWI), and dynamic contrast-enhanced MR imaging (DCE-MRI), has been widely used for detecting and staging prostate cancer [2–7]. DCE-MRI enables the assessment of tumor angiogenesis and has been recommended as an essential component of mp-MRI [8–12]. Currently, the most widely used method to identify tumors on DCE-MRI is to examine focal early enhancement through direct visual assessment. However, the actual kinetics of prostate cancer enhancement are considerably variable and heterogeneous. Absence of early enhancement does not exclude the possibility of malignancy. In addition, the identification of tumors in the transitional zone (TZ) is particularly challenging, because the microvascular density of benign prostatic hyperplasia is similar or even higher than that of prostate tumors and frequently enhances early [13,14]. Considerable efforts have been devoted to determine the enhancement characteristics of prostate cancers, including the use of semiquantitative (pattern analysis of intensity-time curves) and quantitative (compartmental pharmacokinetic modeling) methods, however a consensus regarding the optimal approach for discerning cancerous and benign prostatic tissues is yet to be established.

A semiquantitative analysis of the time-intensity curve of DCE-MRI can demonstrate tissue characteristics by plotting the kinetics of enhancement. Compared with a quantitative analysis, a semiquantitative analysis is less vulnerable to the effect of the arterial input function, B1 inhomogeneities and model selection. A semiquantitative analysis has been applied in prostate DCE-MRI by using traditional average time–intensity curves [14,15]. A histogram analysis is used to quantify the heterogeneity of intratumoral contrast uptake. Perfusion variables can be calculated on a pixel-by-pixel basis to obtain insights into heterogeneity. Studies have demonstrated that histogram parameters can be potential biomarkers for monitoring therapeutic responses and differentiating between cancerous and benign tissues [16,17,18]. In the present study, we conducted a histogram analysis of DCE-MRI time course data to distinguish between benign prostate tissues and tumors in the TZ and peripheral zone (PZ).

## Materials and methods

This retrospective study was approved by the Institutional Review Board (IRB) of Taipei Veterans General Hospital, and methods were performed in accordance with approved guidelines and regulations. The IRB waived the mandate for obtaining informed consent from patients, and the manuscript contains no information or image that can lead to the identification of a study patient.

### Study population

Patients who were proven to have prostate cancer and underwent 3.0-Tesla (T) MR imaging(MRI) between August 2013 and January 2015 were recruited in the present study. MRI indications included preoperative staging for systemic transrectal ultrasound-guided (TRUS) biopsy-confirmed prostate cancer, and survey MRI before targeted TRUS biopsy with MRI–TRUS fusion. After receiving approval from the IRB, the MR images of these patients were retrieved from the picture archiving and communication system and reviewed retrospectively. MR images containing severe motion and susceptibility artifacts and those not adhering to the Prostate Imaging Reporting and Data System 2015 version 2 (PI-RADS v2) were excluded [19]. Two uroradiologists (one with >10 years of experience and the other with 2 years of experience in prostate MRI) reviewed the images together and consensually identified tumors, selecting regions of interest (ROIs) for the subsequent histogram analysis.

### MR image acquisition

MRI was performed on a 3.0-T MRI unit (GE Medical Systems, Milwaukee, WI, USA) by using a body coil for transmission and a four-coil phased array for reception. To reduce motion artifacts in images, peristalsis was suppressed through intramuscular administration of hyoscine butylbromide (Buscopan). Transverse T_2_-weighted fast spin-echo images (T_2_WI) were acquired using the following parameters: repetition time/echo time: 3066–6648/58–98 ms; echo train length: 25; flip angle: 111°; slice thickness: 3 mm; interslice gap: 3 mm; matrix size: 320 × 224; pixel size: 0.35 × 0.35 mm; and number of slices: 30. Axial diffusion-weighted single-shot echo-planar imaging (DWI) sequences were obtained using the following parameters: sensitivity encoding (SENSE)-DWI: 8500/minimum; matrix size: 128 × 128; field of view: 16 × 16 cm; number of excitations: 4; slice thickness/gap: 3/0 mm; b-factor values: 0 and 1000 s/mm^2^ for the three directions of a gradient; and SENSE reduction factor: 2. The corresponding apparent diffusion coefficient (ADC) map was subsequently obtained. DCE-MRI images were acquired using a T_1_-weighted three-dimensional spoiled gradient sequence with the following parameters: repetition time: 2.87–2.95 ms; echo time: 1.38–1.4 ms; flip angle: 12°; section thickness: 3.2 mm; interslice gap: 1.6 mm; field of view: 24×24 cm and number of slices: 42. A total of 25 dynamic phases were obtained with a temporal resolution of 9.96–11.62 s, which was set to the minimum time required for scanning the whole volume of interest. The acquisition time of dynamic scans was at least 5 min. In addition, at the beginning of the fourth measurement, 0.1 mmol/kg of gadodiamide (Magnevist, Schering AG, Berlin, Germany) was intravenously administered followed by a 20-mL saline flush at 2.5 mL/s.

### Image analysis

#### Tumor localization

In the MR report, tumor locations were assigned according to the sector map of PI-RADS v2. For patients who underwent retropubic radical prostatectomy (RRP), tumor locations were confirmed through pathological examinations and designated according to the sector map of PI-RADs V2. Only one lesion on MRI, which was usually the most prominent one, was selected as the target for analysis for each patient. The existence and extent of lesions were confirmed by evaluating the corresponding pathological specimen. For all patients who did not undergo RRP, the malignancy of lesions was confirmed through targeted biopsy. The Gleason score of each lesion was recorded.

#### Selection of ROIs

**Fig 1:**
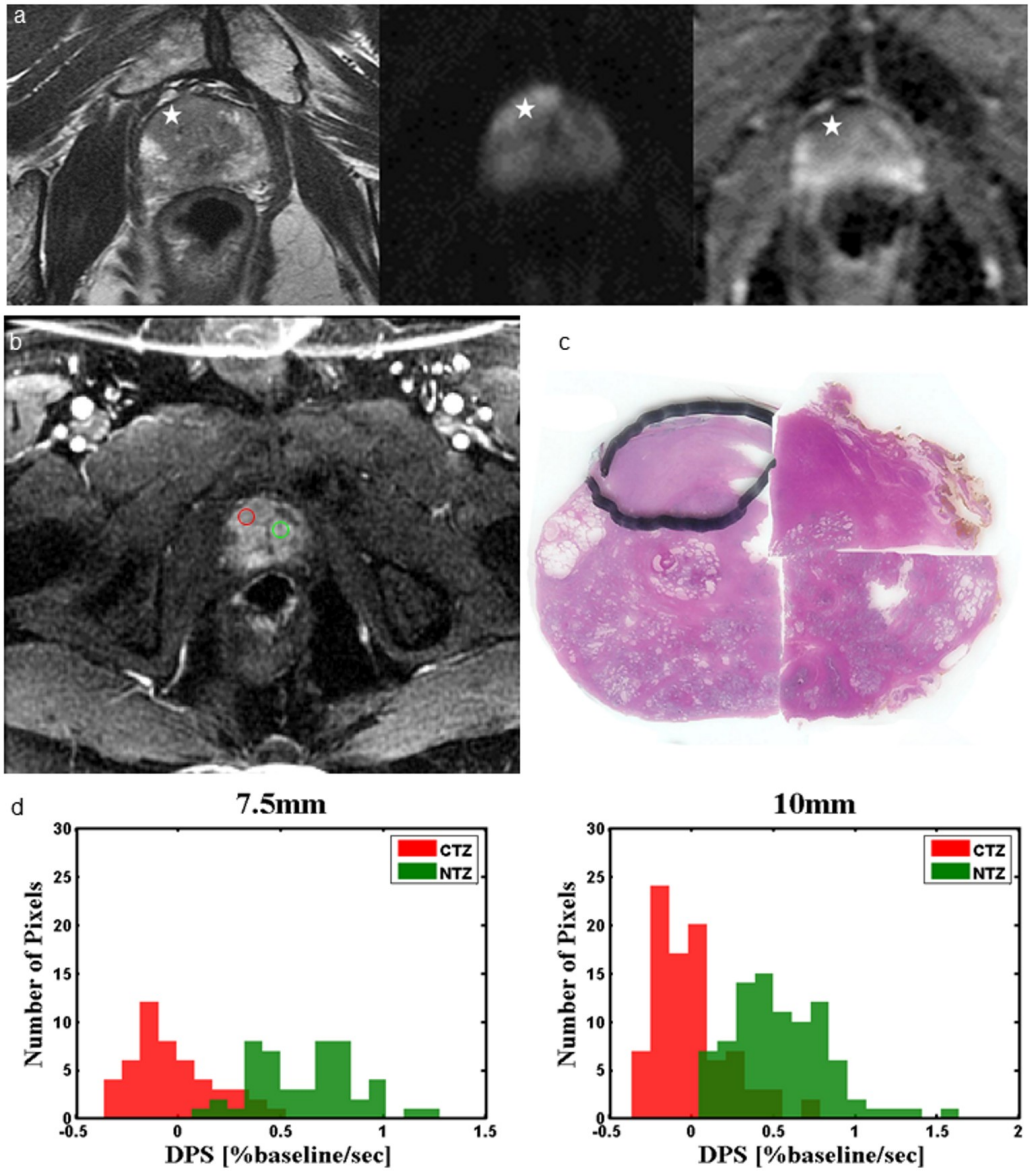
Example of the selected ROI. A 57-year-old patient with elevated PSA levels and previous negative systematic TRUS biopsy. (a) 3T mp-MRI (from left to right: T_2_-weighted fast spin-echo imaging, DWI, and ADC map) demonstrates an area suspected to be cancerous (stars), which was confirmed by performing targeted TRUS biopsy. (b) ROI selection was performed on the basis of corresponding DCE-MRI for CTZ (red circle) and NTZ (green circle). (c) The pathological slice at the corresponding level was correlated to confirm the representativeness of the selected ROI (circled by a marker). (d) The histogram at different diameters of the ROI of CTZ and NTZ.

The uroradiologists used Multi-Image Analysis GUI (http://ric.uthscsa.edu/mango/) software [20] to select circular ROIs from mp-MRI data. ROIs were selected for the normal PZ (NPZ), normal TZ (NTZ), cancer in the PZ (CPZ), and cancer in the TZ (CTZ). To obviate the effect of ROI size on the results of the histogram analysis, circular ROIs with a fixed diameter (7.5 and 10 mm) were obtained for each lesion, and the diameter of ROIs was smaller than the size of the lesion. Within the lesion, the region with the maximum contrast enhancement under visual inspection on DCE-MRI was selected. To ensure the representativeness and independence of selected ROIs, only two ROIs in the same scan plane (one malignant and one benign) were selected for each patient. An example of the selected ROI is depicted in Fig 1a–1c. To remain equidistant from the edge and center of an ROI, circular ROIs were selected.

#### Analysis of DCE-MRI time curve characteristics

**Fig 2:**
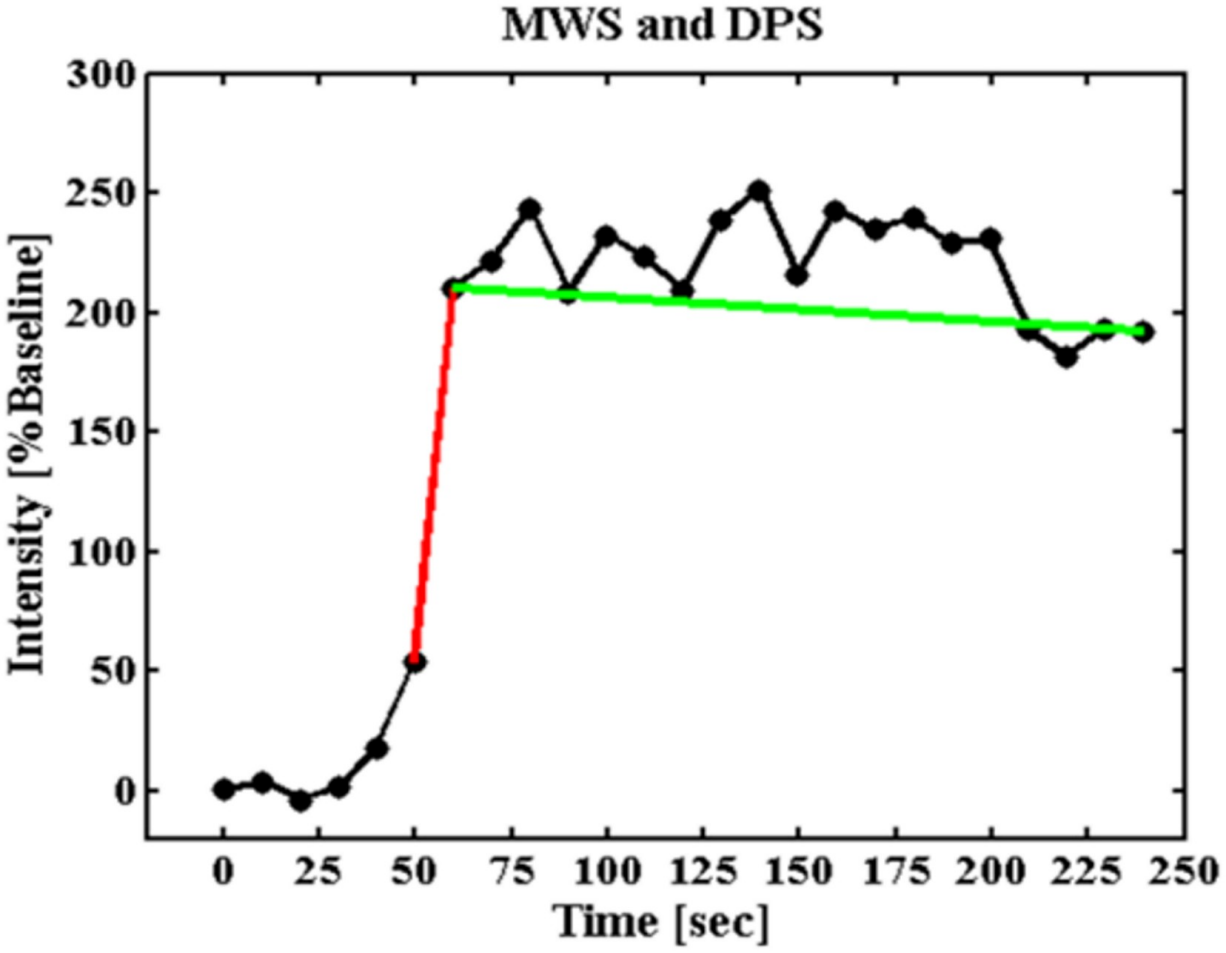
Maximum difference wash-in slope and a delay phase slope are shown during a time course from a single voxel. The wash-in slope (MWS, red line) and the delay phase slope (DPS, green line).

We established a model-free parameter to describe the characteristics of the contrast agent wash-in pattern during early and delay phases in each voxel. To establish the inflow time point of the contrast agent, the intensity of the entire image was calculated. The onset of a signal increase was defined as the inflow time of the contrast agent. The maximum wash-in slope (MWS) was calculated using the maximum difference between two sequential time points, and the timing of the slope was restricted to 30 s after the inflow time. The delay phase washout slope (DPS) was based on intensities from the terminal point of the MWS to the last time point of the dynamic acquisition (Fig 2). For the histogram analysis, the parameters of the MWS and DPS within selected circular ROIs were calculated (Fig 1d). All ROIs were analyzed using an in-house software (Matlab, Mathworks, Natick, MA, USA).

#### Histogram analysis

Histogram parameters were the mean, standard deviation (SD), coefficient of variation (CV), kurtosis, skewness, interquartile range (IQR), percentile (P10, P25, P75, P90, and P90P10), and modified full width at half-maximum (mFWHM). The mFWHM is a quantitative measure used to evaluate the histogram width [17]. The range of the histogram is the difference between the maximum and minimum slope of a sample.

### Statistical analyses

Differences between the histogram parameters of cancerous and benign tissues with regard to different diameters of ROIs were assessed using the nonparametric Wilcoxon signed rank test for paired data. The relationship between the histogram parameters and Gleason score was analyzed using Spearman correlation. The histogram parameters that significantly differed were evaluated using receiver operating characteristic (ROC) curve analysis for determining the diagnosis performance. The optimal threshold value was determined using the Youden index, which indicated diagnostic sensitivity, specificity and accuracy. All statistical analyses were performed using SPSS version 22 (IBM Corp., Armonk, NY, USA). A *P* value of <0.05 was considered statistically significant for all tests.

## Results

A total of 30 patients with a median age of 65.5 (range: 55–78) years and a median prostate-specific antigen (PSA) level of 12.945 (range: 5.5–57.5) ng/mL were recruited for additional imaging analyses. Of these, 12 were scanned for preoperative staging after systemic TRUS biopsy-confirmed prostate cancer and underwent subsequent RRP. A total of 18 patients were scanned due to increased PSA levels, with at least one negative systematic biopsy; their tumors were proved through targeted TRUS biopsy. The clinical and pathological characteristics of recruited patients are summarized in Table 1.

**Table 1:** Summary of clinical and pathologic characteristics.

Parameter	Value	
Median age at patients (y)*	65.5 (55–78)	
Median prostate-specific antigen level (ng/ml)*	12.945 (5.5–57.5)	
Tumor location	Transitional zone (n = 16)	peripheral zone (n = 14)
Clinical stage at prostatectomy		
T2b	2	1
T2c	4	2
T3a	8	6
T3b	1	3
Radiation therapy	1	2
Gleason score		
3+3	2	0
3+4	7	7
4+3	7	3
4+4	0	2
4+5	0	2

*Data are medians with ranges in parentheses

A total of 60 ROIs with a diameter of 7.5 mm were selected in pairs in 30 patients, including 16, 16, 14, and 14 in the CTZ, NTZ, CPZ, and NPZ, respectively. The median tumor width was 12.5 (range: 10–27) mm in TZ and 10 (range: 10–26) mm in the PZ.

### Comparison of histogram parameters between cancerous and normal tissues

Table 2 summarizes differences in the MWS and DPS between the CTZ and NTZ. In the MWS, CV for both ROIs of 7.5 and 10 mm(*P* = 0.034 and *P* = 0.004, respectively) and P10 for an ROI of 10 mm (*P* = 0.044) were the only parameters that differed significantly. The CV of the NTZ was higher than that of the CTZ. In contrast to the MWS, many parameters exhibited significant differences in the DPS. The mean, P25, P50, P75, and P90 of the DPS were significantly smaller in the CTZ than in the NTZ, regardless of the ROI diameter (*P* <0.001–0.016); the most significant difference was observed in P50 for ROIs of 7.5 and 10 mm (*P* <0.001). For SD, a significant difference was observed in only the ROI of 7.5 mm. For skewness and P10, a significant difference was observed in only the ROI of 10 mm.

**Table 2:** Differences in the MWS and DPS histogram parameters between CTZ and NTZ with different diameters of the ROI.

	MWS 7.5 mm			MWS 10 mm			DPS 7.5 mm			DPS 10 mm		
Parameter	CTZ(n = 16)	NTZ(n = 16)	*P-* value	CTZ(n = 16)	NTZ(n = 16)	*P-* value	CTZ(n = 16)	NTZ(n = 16)	*P-* value	CTZ(n = 16)	NTZ(n = 16)	*P-* value
Mean	9.26(3.58)	7.69(2.94)	0.193	8.88(3.13)	7.21(2.65)	0.107	0.15(0.24)	0.43(0.27)	0.001**	0.17(0.24)	0.46(0.27)	<0.001***
SD	2.36(0.76)	2.46(0.81)	0.98	2.48(0.81)	2.55(0.87)	0.792	0.18(0.18)	0.37(0.28)	0.007*	0.2(0.05)	0.25(0.12)	0.147
CV	0.27(0.05)	0.34(0.09)	0.034*	0.29(0.05)	0.37(0.09)	0.004**	0.63(2.8)	1.01(1.09)	0.744	-3.67(17.02)	0.69(0.42)	0.744
Kurtosis	2.84(0.75)	3.05(0.9)	0.495	2.8(0.61)	2.94(0.75)	0.231	2.21(1.32)	1.99(1.25)	0.678	3.16(0.78)	2.94(0.78)	0.269
Skewness	0.31(0.45)	0.46(0.46)	0.52	0.28(0.37)	0.47(0.36)	0.171	0.32(0.78)	0.05(0.45)	0.433	0.39(0.52)	-0.04(0.49)	0.004**
IQR	3.31(1.23)	3.32(1.23)	0.98	3.55(1.37)	3.49(1.37)	0.782	0.33(0.2)	0.53(0.69)	0.349	0.27(0.07)	0.35(0.23)	0.216
P10	6.2(2.38)	4.77(2.18)	0.105	5.79(2.27)	4.12(1.92)	0.044*	0.37(1.13)	0.29(0.35)	0.229	-0.07(0.24)	0.12(0.23)	0.01*
P25	7.57(3.12)	5.91(2.44)	0.093	7.05(2.63)	5.36(2.1)	0.074	0.21(0.74)	0.46(0.73)	0.002**	0.04(0.24)	0.29(0.24)	0.001**
P50	9.1(3.74)	7.37(2.97)	0.193	8.72(3.25)	6.93(2.53)	0.093	-0.05(0.6)	0.45(0.33)	<0.001***	0.15(0.25)	0.48(0.29)	<0.001***
P75	10.87(4.2)	9.23(3.45)	0.193	10.6(3.69)	8.84(3.23)	0.144	0.27(0.25)	0.55(0.35)	0.013*	0.3(0.25)	0.64(0.34)	<0.001***
P90	12.38(4.4)	11.08(3.79)	0.404	12.16(4.07)	10.7(3.64)	0.252	0.45(0.22)	0.81(0.61)	0.016*	0.45(0.24)	0.77(0.37)	0.001**
mFWHM	5.88(2.11)	6(2.02)	0.978	5.84(2.02)	6.12(2.15)	0.9	0.44(0.2)	0.6(0.32)	0.056	0.48(0.13)	0.61(0.32)	0.06
P90P10	6.18(2.16)	6.31(2.09)	0.9	6.36(2.27)	6.59(2.16)	0.706	0.44(0.21)	0.47(0.49)	0.441	0.52(0.14)	0.65(0.34)	0.09
Range	10.09(3)	10.86(3.72)	0.782	11.33(3.3)	11.83(3.92)	0.716	0.82(0.47)	0.91(0.61)	0.316	0.95(0.2)	1.11(0.44)	0.08

All values are expressed as the mean (standard deviation), maximum wash-in slope (MWS), delay phase slope (DPS), cancer in the transitional zone (CTZ), and the normal transitional zone (NTZ). The comparsion between tumor and normal tissues for every parameter was performed using the Wilcoxon signed rank test. *P* < 0.05 was considered significant.

*P < 0.05

**P < 0.01

***P < 0.001

Table 3 summarizes differences in the MWS and DPS between the CPZ and NPZ. All the parameters of the MWS exhibited significant differences between the CPZ and NPZ, except for kurtosis and skewness for an ROI of 7.5 mm. Kurtosis and skewness for an ROI of 10 mm were significantly lower in the CPZ than in the NPZ (*P* = 0.03 and *P* = 0.017, respectively), and all the remaining parameters were significantly higher in the CPZ than in the NPZ (*P* < 0.001–0.03). The most significant differences were observed in CV, P10–P90, and mean in ROIs of 7.5 and 10 mm (*P* < 0.001). Not as many parameters as in the MWS showed significant differences in the DPS. The range and SD for an ROI of 10 mm in the DPS differed most significantly (*P* = 0.001 and *P* = 0.008, respectively).

**Table 3:** Differences in the MWS and DPS histogram parameters between CPZ and NPZ with different diameters of the ROI.

	MWS 7.5 mm			MWS 10 mm			DPS 7.5 mm			DPS 10 mm		
Parameter	CPZ(n = 14)	NPZ(n = 14)	*P-* value	CPZ(n = 14)	NPZ(n = 14)	*P-* value	CPZ(n = 14)	NPZ(n = 14)	*P-* value	CPZ(n = 14)	NPZ(n = 14)	*P-* value
Mean	9.37(3.88)	3.96(2.08)	<0.001***	8.82(3.31)	4.14(2.1)	<0.001***	0.12(0.22)	0.26(0.21)	0.123	0.17(0.21)	0.25(0.19)	0.453
SD	2.71(1.1)	1.63(0.57)	0.002**	2.9(1.14)	1.79(0.65)	0.001**	0.19(0.06)	0.15(0.08)	0.038*	0.21(0.07)	0.16(0.08)	0.008**
CV	0.31(0.09)	0.46(0.13)	<0.001***	0.34(0.09)	0.47(0.11)	<0.001**	1.48(1.87)	0.29(2.04)	0.268	1.58(3.17)	1.06(0.72)	0.761
Kurtosis	3.49(2.26)	3.7(1.08)	0.268	3.19(1.17)	3.83(1.61)	0.03*	3.23(1.26)	2.88(0.71)	0.808	3.3(1.13)	3.3(1.11)	0.988
Skewness	0.52(0.64)	0.74(0.53)	0.135	0.52(0.43)	0.78(0.47)	0.017*	-0.03(0.78)	0.33(0.44)	0.241	0.16(0.68)	0.45(0.55)	0.274
IQR	3.5(1.43)	1.96(0.72)	0.001**	3.95(1.73)	2.38(0.98)	0.004**	0.25(0.1)	0.21(0.11)	0.133	0.27(0.11)	0.21(0.12)	0.028*
P10	6.05(2.87)	2.13(1.55)	<0.001***	5.29(2.24)	2.06(1.53)	<0.001***	-0.11(0.23)	0.07(0.15)	0.012*	-0.09(0.22)	0.05(0.12)	0.065
P25	7.49(3.33)	2.86(1.88)	<0.001***	6.63(2.66)	2.83(1.66)	<0.001***	0(0.21)	0.15(0.16)	0.029*	0.03(0.2)	0.13(0.15)	0.157
P50	9.14(3.75)	3.69(2.01)	<0.001***	8.56(3.32)	3.95(2.07)	<0.001***	0.13(0.21)	0.25(0.2)	0.167	0.17(0.21)	0.23(0.19)	0.508
P75	10.99(4.47)	4.82(2.34)	<0.001***	10.58(4.03)	5.21(2.52)	<0.001***	0.25(0.21)	0.36(0.25)	0.302	0.3(0.21)	0.34(0.23)	0.915
P90	13.13(5.38)	6.17(2.65)	<0.001***	12.76(4.86	6.58(2.98)	<0.001***	0.37(0.25)	0.47(0.3)	0.572	0.42(0.23)	0.46(0.28)	0.915
mFWHM	6.61(3.02)	3.73(1.34)	0.001**	6.77(2.74)	4.23(1.67)	0.002**	0.45(0.17)	0.38(0.21)	0.065	0.46(0.16)	0.38(0.18)	0.035*
P90P10	7.08(3.17)	4.05(1.45)	0.001**	7.48(3.15)	4.52(1.7)	0.002**	0.48(0.18)	0.4(0.21)	0.075	0.51(0.17)	0.41(0.19)	0.028*
Range	11.86(4.1)	7.26(2.52)	0.001**	13.31(4.68)	8.63(2.8)	0.002**	0.79(0.26)	0.67(0.33)	0.094	1.01(0.34)	0.77(0.34)	0.001**

All values are expressed as the mean (standard deviation), maximum wash-in slope (MWS), delay phase slope (DPS), cancer in the peripheral zone (CPZ), and normal peripheral zone (NPZ). A comparsion between tumor and normal tissues for every parameter was performed using the Wilcoxon signed rank test. *P* < 0.05 was considered significant.

*P < 0.05

**P < 0.01

***P < 0.001

### Correlation of histogram parameters with the Gleason score

We correlated the significantly different histogram parameters with the Gleason score of tumors (Table 4). A total of 2 and 14 tumor lesions in the TZ had Gleason scores of 6 [3+3] and 7 [3+4, 4+3], respectively. A total of 10, 2, and 2 tumor lesions in the PZ had Gleason scores of 7 [3+4, 4+3], 8 [4+4], and 9 [4+5], respectively. In the TZ, the skewness of the DPS is the only parameter which was significantly correlated with the Gleason score (*ρ* = 0.533). In the PZ, none of the parameters showed significant correlation with the Gleason score.

**Table 4:** The Spearman correlation coefficient for correlation of histogram parameter with Gleason score.

	TZ			PZ	
Parameter	*ρ*	*P*-value	Parameter	*ρ*	*P*-value
**MWS**			**MWS**		
CV	-0.144	0.297	Mean	0.166	0.286
P10	0.123	0.325	SD	0.144	0.312
**DPS**			CV	-0.072	0.403
Mean	-0.041	0.44	IQR	0.044	0.44
Skewness	0.533	0.017*	P10	0.232	0.212
P10	-0.041	0.44	P25	0.088	0.382
P25	-0.164	0.272	P50	0.088	0.382
P50	-0.103	0.352	P75	0.077	0.396
P75	-0.041	0.44	P90	0.188	0.26
P90	0	0.5	mFWHM	-0.022	0.47
			P90P10	0.11	0.485
			Range	0.077	0.396

TZ, transitional zone; PZ, peripheral zone; MWS, maximum wash-in slope; DPS, delay phase slope. *P* < 0.05 was considered significant.

*P < 0.05

### ROC curve analysis of histogram parameters

**Fig 3:**
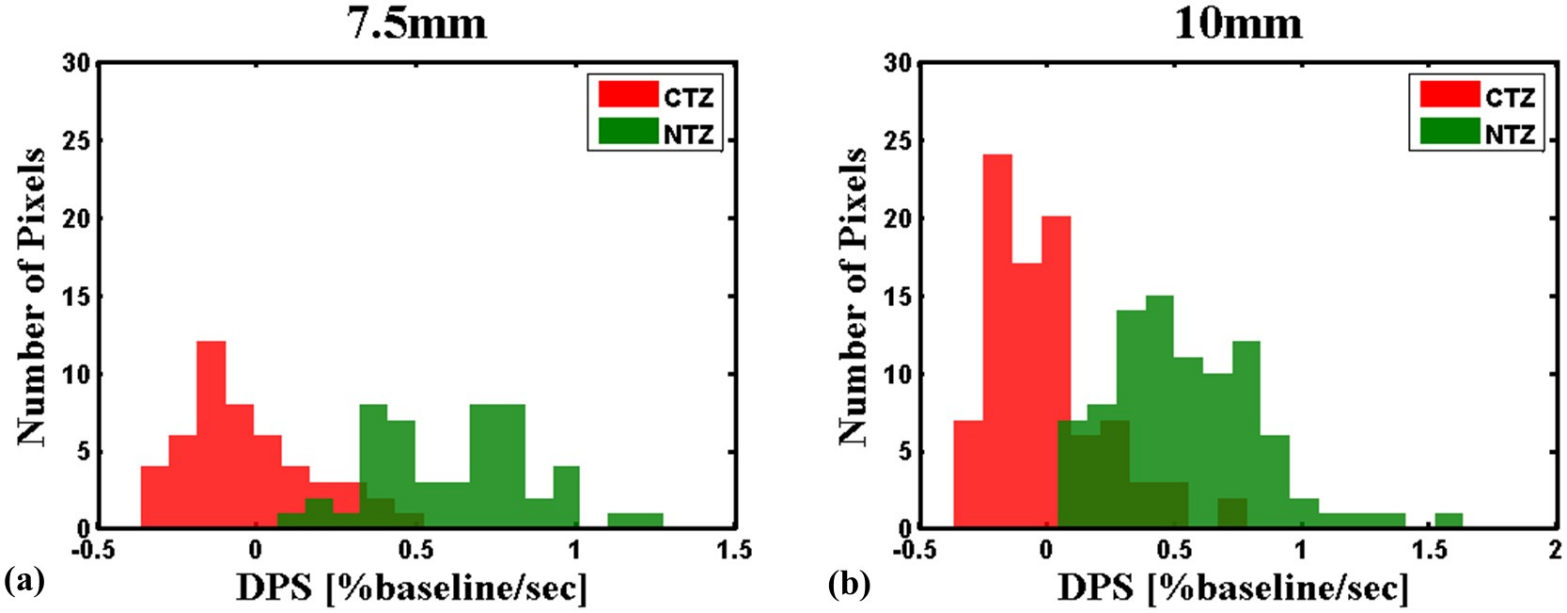
Comparison of the ROC curves of histogram parameters in the differentiation of tumors with maximum difference wash-in and delay phase slopes. The parameters of the MWS (red lines) and those of the DPS (blue lines) in (a) the transitional zone and (b) the peripheral zone. Note the AUC of the parameters of the DPS (blue lines) are generally larger than those of the MWS (red lines) in (a), while contrary in (b).

The ROC curve analysis was performed for the most significant histogram parameters. Table 5 lists the threshold values, area under the curve (AUC), sensitivity, specificity, and, accuracy of the histogram parameters. An ROI of 10 mm exhibited more significant differences compared with an ROI of 7.5 mm; therefore, the ROC curve analysis was performed for an ROI of 10 mm from cancerous and benign tissues (Table 5, Fig 3). In the TZ, CV, mean, P10, P25, P50, P75, P90, and skewness both in the MWS and DPS were selected for the ROC curve analysis. AUC values were between 0.688 and 0.822, with CV of the MWS and P50 of the DPS being the highest (0.82 and 0.822, respectively). The sensitivity of these parameters was generally low (approximately 62%), whereas the specificity was high (approximately more than 80%). Compared with the MWS, most parameters of the DPS had higher AUC values, except for the CV (Fig 3a).

**Table 5:** The threshold values, AUC, sensitivity, specificity and accuracy of the histogram parameters (10 mm of ROI).

Transitional Zone						Peripheral Zone					
Parameters	Threshold	AUC	Sensitivity(%)	Spcificity(%)	Accuracy(%)	Parameters	Threshold	AUC	Sensitivity(%)	Spcificity(%)	Accuracy(%)
**MWS**						**MWS**					
CV	0.355	0.82	93.8	62.5	78.13	Mean	4.98	0.893	92.9	78.6	85.7
P10	5.685	0.688	56.3	81.3	68.75	CV	0.375	0.827	71.4	85.7	78.5
**DPS**						P10	2.835	0.893	92.9	78.6	85.7
Mean	0.185	0.799	62.5	87.5	75	P25	3.24	0.898	92.9	71.4	82.1
Skewness	0.265	0.73	62.5	81.3	76.7	P50	4.78	0.883	92.9	78.6	85.7
P10	0.025	0.742	81.3	68.8	75	P75	6.235	0.87	85.7	78.6	78.6
P25	0.055	0.801	62.5	87.5	75	P90	10.56	0.865	71.4	92.9	82.1
P50	0.13	0.822	62.5	93.8	78.13	**DPS**					
P75	0.245	0.803	56.3	93.8	75	SD	0.105	0.676	100	35.7	67.9
P90	0.535	0.768	75	68.8	71.9	Range	0.57	0.681	92.9	35.7	64.3

MWS, maximum wash-in slope; DPS, delay phase slope; AUC, area under the curve.

In the PZ, the ROC curve analysis was performed for mean, CV, P10, P25, P50, P75, P90, SD, and range both in the MWS and DPS, with the AUC value being between 0.676 and 0.893. The AUC values of the parameters in the MWS were generally high (between 0.827 and 0.898; Fig 3b). Some of the parameters in the DPS showed satisfactory performance with the AUC value between 0.638 and 0.681.

## Discussion

The traditional semiquantitative analysis of DCE-MRI for prostate cancer involves the use of average time-intensity curves [14,15]. For each selected ROI, time-signal intensity curves normalized to baseline were generated, and a signal intensity and mean gradient method were established for calculating signal changes between the inflow time and time required for the maximum enhancement of the contrast agent. However, this approach has several limitations. First, the averaging process may obscure the information regarding vascular heterogeneity. Second, confounding factors and noise on time can interfere in the measurement of the wash-in slope. Third, the time required for the maximum enhancement cannot be observed when the signal consistently increases or remains steady [21]. Moreover, noise spikes in the plateau region of a time course may be misidentified as the maximum enhancement, resulting in miscalculations in the wash-in slope.

To overcome the aforementioned limitations, we proposed a simple and robust method of extracting two semiquantitative parameters for describing the contrast agent behavior. We defined the maximum signal difference between two sequential time points as the MWS and restricted the timing of the slope to 30 s after the inflow time for preventing the interference of noise spikes [22]. After the terminal point of the MWS, we observed a gradual increase in signals until the maximum enhancement of the contrast agent or the end of the time course. To describe this part of the time course, we defined the DPS as the slope between the terminal time point of the MWS and the end of the time course. Theoretically, the DPS consists of the delayed wash-in and washout time points of the contrast agent. The MWS and DPS revealed distinct physiological information. In the early stage following the entry of the contrast agent, increases in signals depend mainly on the permeability and surface area of vessel walls. The maximum difference between consecutive signals (i.e., MWS) occurs when the concentration gradient reaches the maximum across the vessel wall. After the terminal point of the MWS, the concentration gradient decreases because of the increase in the concentration of the extravascular extracellular space (EES). The increase in the EES concentration should be inversely proportional to the EES. The DPS may reflect the characteristics of the EES in the prostate interstitium.

In this study, a histogram analysis of the MWS and DPS was performed to quantify the heterogeneity of tumors [23]. The study findings revealed that the histogram parameters capable of distinguishing between benign and cancerous tissues were different in the PZ and TZ. In the PZ, most parameters of the MWS showed significant differences between cancerous and normal tissues. The results are consistent with those of previous studies that demonstrated that prostate cancer is often found to enhance rapidly to a greater extent than benign PZ tissues, as well as to show a more rapid clearance [22,24]. This indicates that tumors in the PZ can be more easily diagnosed by visual identification.

In the TZ, most parameters of the MWS revealed no significant differences between benign and cancerous tissues. The statistical nonsignificance of other MWS parameters between cancerous and benign tissues may be due to a similar wash-in pattern [24]. The benign proliferative process causes an increase in the microvascular density similar to that observed in cancer [14]. CV was the only significant MWS parameter in both sizes of the ROI. The CV in the CTZ was smaller than that in the NTZ, indicating that the variation in the MWS was lower in cancerous tissues than in benign tissues. By contrast, many histogram parameters of the DPS differed significantly between cancerous and benign tissues in the TZ. Previous studies have reported that the washout slope of DCE-MRI can differentiate between benign and malignant prostate tumors [25,26]. However, prostate cancer was not separately analyzed in the PZ and TZ. In our study, the histogram analysis was conducted to evaluate PZ and TZ tumors separately. With this strategy, the AUC of significant parameters in the PZ and TZ could reach 0.865–0.898 and 0.688–0.822, respectively (Table 5).

We demonstrated the effect of the diameters of ROIs on the histogram parameters. The mean, P25, P50, P75, and P90 of the DPS exhibited a significant difference in the TZ, regardless of the diameter of the ROI. The SD of the DPS showed a significant difference only in an ROI of 7.5 mm, whereas skewness and P10 showed significant differences only in an ROI of 10 mm. Although the sample size is yet too small for making a conclusion, the results implied that the SD, skewness, and P10 may be susceptible to changes in ROI diameters in the TZ. The increased heterogeneity in an ROI with a larger diameter may affect these parameters. Our results confirm that the diameters of ROIs must be considered when performing a histogram analysis. Therefore, instead of selecting a segment of the whole tumor, a reasonable strategy is to sample the most significantly enhanced area within the tumor as the ROI. An ROI with a diameter of 10 mm is the most appropriate size because most parameters may distinguish between benign and cancerous tissues for an ROI of 10 mm.

The results of this study showed a weak correlation of the Gleason score with the histogram parameters. The skewness of the DPS is the only significant parameter that correlated with the Gleason score in the TZ. In the PZ, none of the parameters showed significant correlation with the Gleason score. A small patient size and selection bias may, in part, explain the weak correlation of the Gleason score with the histogram parameters [14]. The numbers of tumors with Gleason scores of 6 in the PZ and 9 in the TZ were not included. Variations in signal enhancement and spatial and temporal resolutions may affect time course characteristics. A previous study reported that a better temporal resolution may be a factor affecting the correlation between DCE-MRI parameters and the Gleason score [27]. The temporal resolution in our study was approximately 9.96–11.62 s, which may have also affected the correlation between the histogram parameters and Gleason score.

Our study has some limitations. First, the small sample size could have been influenced by selection and verification biaes. Second, we did not distinguish between various NTZ types in patients. The enhancement pattern may differ between stromal and glandular hyperplasia. Additional studies analyzing the time course pattern and biopsy confirmation of prostate cancer are warranted. Third, this study is the first step in the identification of effective quantitative image parameters, and it demonstrates the benefit of DCE-MR image analysis. Future studies are warranted to investigate more combined methods to increase the detection accuracy of prostate cancer [5–8].

In conclusion, we proposed a histogram analysis method for analyzing the time course of prostate DCE-MRI and demonstrated its potential capability in the diagnosis of prostate cancer. Different histogram parameters of the MWS and DPS for efficiently distinguishing between cancerous and benign prostatic tissues should be applied in the TZ and PZ. Based on preliminary results, generating the parametric imaging of the EWS and DPS of prostate DCE-MRI may be the reasonable next step.

## Supporting information

S1 Table The raw data of the histogram analysis. All values are expressed as maximum wash-in slope (MWS), delay phase slope (DPS), cancer in the transitional zone (CTZ), the normal transitional zone (NTZ), cancer in the peripheral zone (CPZ), normal peripheral zone (NPZ).
(XLSX)

S1 Fig IRB approval letter. (PDF)

S2 Fig English editing certificate. (PDF)

## References

[1] Siegel RL, Fedewa SA, Miller KD, Goding-Sauer A, Pinheiro PS, Martinez-Tyson D, et al Cancer statistics for Hispanics/Latinos, 2015. Ca-Cancer J Clin. 2015 Nov-Dec;65(6):457–80. 10.3322/caac.21314 26375877

[2] Kim JY, Kim SH, Kim YH, Lee HJ, Kim MJ, Choi MS. Low-Risk Prostate Cancer: The Accuracy of Multiparametric MR Imaging for Detection. Radiology. 2014 5;271(2):435–44. 10.1148/radiol.13130801 24484061

[3] Hambrock T, Vos PC, Hulsbergen-van de Kaa CA, Barentsz JO, Huisman HJ. Prostate cancer: computer-aided diagnosis with multiparametric 3-T MR imaging—effect on observer performance. Radiology. 2013 2;266(2):521–30. 10.1148/radiol.12111634 23204542

[4] Turkbey B, Pinto PA, Mani H, Bernardo M, Pang Y, McKinney YL, et al Prostate cancer: value of multiparametric MR imaging at 3 T for detection—histopathologic correlation. Radiology. 2010 4;255(1):89–99. 10.1148/radiol.09090475 20308447 PMC2843833

[5] Fusco R, Sansone M, Granata V, Setola SV, Petrillo A. A systematic review on multiparametric MR imaging in prostate cancer detection. Infect Agent Cancer. 2017 10 30;12:57 10.1186/s13027-017-0168-z eCollection 2017. 29093748 PMC5663098

[6] Petrillo A, Fusco R, Setola SV, Ronza FM, Granata V, Petrillo M, et al Multiparametric MRI for prostatecancer detection: performance in patients with prostate-specific antigen values between 2.5 and 10 ng/mL. J Magn Reson Imaging. 2014 5;39(5):1206–12. 25006636 10.1002/jmri.24269

[7] Fusco R, Sansone M, Petrillo M, Setola SV, Granata V, Botti G, et al Multi-parametric MRI for prostate cancer detection: Preliminary results on quantitative analysis of dynamic contrast enhancedimaging, diffusion-weighted imaging and spectroscopy imaging. Magn Reson Imaging. 2016 9;34(7):839–45. 10.1016/j.mri.2016.04.001 Epub 2016 Apr 9. 27071309

[8] Perdona S, Di Lorenzo G, Autorino R, Buonerba C, De Sio M, Setola SV, et al Combined magnetic resonance spectroscopy and dynamiccontrast-enhanced imaging for prostate cancer detection. Urol Oncol. 2013 8;31(6):761–5. 10.1016/j.urolonc.2011.07.010 Epub 2011 Sep 9. 21906966

[9] Jackson A, O’Connor JP, Parker GJ, Jayson GC. Imaging tumor vascular heterogeneity and angiogenesis using dynamic contrast-enhanced magnetic resonance imaging. Clin Cancer Res. 2007 6 15;13(12):3449–59. 10.1158/1078-0432.CCR-07-0238 17575207

[10] Fan X, Medved M, River JN, Zamora M, Corot C, Robert P, et al New model for analysis of dynamic contrast-enhanced MRI data distinguishes metastatic from nonmetastatic transplanted rodent prostate tumors. Magn Reson Med. 2004 3;51(3):487–94. 10.1002/mrm.10737 15004789

[11] Isebaert S, De Keyzer F, Haustermans K, Lerut E, Roskams T, Roebben I, et al Evaluation of semi-quantitative dynamic contrast-enhanced MRI parameters for prostate cancer in correlation to whole-mount histopathology. Eur J Radiol. 2012 3;81(3):e217–22. 10.1016/j.ejrad.2011.01.107 21349667

[12] Vos EK, Litjens GJ, Kobus T, Hambrock T, Hulsbergen-van de Kaa CA, Barentsz JO, et al Assessment of prostate cancer aggressiveness using dynamic contrast-enhanced magnetic resonance imaging at 3T. Eur Urol. 2013 9;64(3):448–55. 10.1016/j.eururo.2013.05.045 23751135

[13] Van Niekerk CG, Witjes JA, barentsz JO, Van der Laak JA, Hulsbergen-van de Kaa CA. Microvascularity in transition zone prostate tumors resembles normal prostatic tissue. Prostate. 2013 4;73(5):467–75. 10.1002/pros.22588 22996830

[14] Padhani AR, Gapinski CJ, Macvicar DA, Parker GJ, Suckling J, Revell PB, et al Dynamic contrast enhanced MRI of prostate cancer: correlation with morphology and tumour stage, histological grade and PSA. Clin Radiol. 2000 2;55(2):99–109. 10.1053/crad.1999.0327 10657154

[15] Noworolski SM, Henry RG, Vigneron DB, Kurhanewicz J. Dynamic contrast-enhanced MRI in normal and abnormal prostate tissues as defined by biopsy, MRI, and 3D MRSI. Magn Reson Med. 2005 2;53(2):249–55. 10.1002/mrm.20374 15678552

[16] Just N. Improving tumour heterogeneity MRI assessment with histograms. Br J Cancer. 2014 12 9;111(12):2205–13. 10.1038/bjc.2014.512 25268373 PMC4264439

[17] Peng SL, Chen CF, Liu HL, Lui CC, Huang YJ, Lee TH, et al Analysis of parametric histogram from dynamic contrast-enhanced MRI: application in evaluating brain tumor response to radiotherapy. NMR Biomed. 2013 4;26(4):443–50. 10.1002/nbm.2882 23073840

[18] Yang X, Knopp MV. Quantifying Tumor Vascular Heterogeneity with Dynamic Contrast-Enhanced Magnetic Resonance Imaging: A Review. J Biomed Biotechnol. 2011;2011:732848 10.1155/2011/732848 21541193 PMC3085501

[19] American Collage of Radiology. PI-RADS v2.2015; http://www.acr.org/Quality-Safety/Resources/PIRADS(2015)

[20] Multi-Image Analysis GUI 2013; http://ric.uthscsa.edu/mango/

[21] Zelhof B, Lowry M, Rodrigues G, Kraus S, Turnbull L. Description of magnetic resonance imaging-derived enhancement variables in pathologically confirmed prostate cancer and normal peripheral zone regions. BJU Int. 2009 9;104(5):621–7. 10.1111/j.1464-410X.2009.08457.x 19281464

[22] Preziosi P, Orlacchio A, Di Giambattista G, Di Renzi P, Bortolotti L, Fabiano A, et al Enhancement patterns of prostate cancer in dynamic MRI. Eur Radiol. 2003 5;13(5):925–30. 12695811 10.1007/s00330-002-1703-9

[23] Just N. Improving tumour heterogeneity MRI assessment with histograms. British Journal of Cancer. 2014 12 9;111(12):2205–13. 10.1038/bjc.2014.512 Epub 2014 Sep 30. 25268373 PMC4264439

[24] Rouviere O, Raudrant A, Ecochard R, Colin-Pangaud C, Pasquiou C, Bouvier R, et al Characterization of time-enhancement curves of benign and malignant prostate tissue at dynamic MR imaging. Eur Radiol. 2003 5;13(5):931–42. 12695812 10.1007/s00330-002-1617-6

[25] Chen YJ, Chu WC, Pu YS, Chueh SC, Shun CT, Tseng WY. Washout grasient in dynamic contrast-enhanced MRI is associated with tumor aggressiveness of prostate cancer. J Magn Reson Imaging. 2012 10;36(4):912–9. 10.1002/jmri.23723 22711415

[26] Engelbrecht MR, Huisman HJ, Laheij RJ, Jager GJ, van Leenders GJ, Hulsbergen-Van De Kaa CA, et al Discrimination of prostate cancer from normal peripheral zone and central gland tissue by using dynamic contrast-enhanced MR imaging. Radiology. 2003 10;229(1):248–54. 10.1148/radiol.2291020200 12944607

[27] Peng Y, Jiang Y, Yang C, Brown JB, Antic T, Sethi I, et al Quantitative analysis of multiparametric prostate MR images: differentiation between prostate cancer and normal tissue and correlation with Gleason score—a computer-aided diagnosis development study. Radiology. 2013 6;267(3):787–96. 10.1148/radiol.13121454 Epub 2013 Feb 7. 23392430 PMC6940008

