# Supplementary material for: Histogram analysis of prostate cancer on dynamic contrast-enhanced magnetic resonance imaging: A preliminary study emphasizing on zonal difference
Source: PLoS One. 2019 Feb 12;14(2):e0212092. doi: 10.1371/journal.pone.0212092 (PMC6372178; doi:10.1371/journal.pone.0212092)
Supplement: S1 Fig — (PDF) [file pone.0212092.s002.pdf]

臺 北 榮 民 總 醫 院
TAIPEI VETERANS GENERAL HOSPITAL
201 SHIH-PAI ROAD, SEC. 2
TAIPEI, TAIWAN 11217
REPUBLIC OF CHINA
TEL: (886)-2-2871-2121(30 LINES)

同 意 臨 床 試 驗 證 明 書

查本院放射線部沈書慧醫師主持之「以 wash-in slope 直方圖區分良性與惡性攝護腺組織」（本院 IRB 編號：2014-11-005CC）臨床試驗案，業經本院 103 年 11 月 26 日人體試驗委員會(三)審查通過，有效期限至 104 年 11 月 25 日，特此證明。

計畫主持人須於到期前2個月至6週（至少前6週）提出持續審查之申請，本案須經本院人體試驗委員會通過後，方可繼續執行。（凡需送衛生福利部審核之計畫案件，須取得衛生福利部審核同意函後方可開始執行）

臺北榮民總醫院
人體試驗委員會
主任委員
何善台

中 華 民 國 1 0 3 年 1 2 月 1 7 日

Dec 17, 2014

To Whom It May Concern:

RE: To differentiate normal and malignant tissue by wash-in slope histogram in prostate gland 整合型總計畫名稱 Advanced Technique for Quantitative Prostate DCE-MRI

Principal Investigator: Shu-Huei Shen, M.D.

Co-Investigator: Hsiao-Jen Chung, M.D., Tzu-Ping Lin, M.D.

Version date of documents:

1. Protocol : Version 1, Date: Oct 28, 2014
2. Synopsis: Version 1, Date: Oct 28, 2014
3. Informed Consent Form: Waived

VGHIRB No.: 2014-11-005CC

According to the written operating procedures, GCP, and the applicable regulatory requirements, this study project is approved by the Institutional Review Board of Taipei Veterans General Hospital. The board is organized under, and operates according to International Conference on Harmonisation (ICH) / WHO Good Clinical Practice (GCP) and the applicable laws and regulations.

This approval is valid for 1 year till Nov 25, 2015. The principal investigator is required to submit the application for extension 6 weeks before the expiration date. (If indicated by the regulations and laws, this project should be taken after the approval of Ministry of Health and Welfare, R.O.C.)

Shung-Tai Ho, M.D.
Chairman
Institutional Review Board
Taipei Veterans General Hospital
Taiwan, R.O.C.
