# Supplementary material for: Histogram analysis of prostate cancer on dynamic contrast-enhanced magnetic resonance imaging: A preliminary study emphasizing on zonal difference
Source: PLoS One. 2019 Feb 12;14(2):e0212092. doi: 10.1371/journal.pone.0212092 (PMC6372178; doi:10.1371/journal.pone.0212092)
Supplement: S2 Fig — (PDF) [file pone.0212092.s003.pdf]

WALLACE

# Wallace Academic Editing

## English Editing Certificate

This certifies that the paper **Histogram analysis of prostate cancer on dynamic contrast-enhanced magnetic resonance imaging: A preliminary study emphasizing on zonal difference** has been edited by Paula Bensley on December 15, 2018 and is considered to be improved in grammar, punctuation, spelling, verb usage, sentence structure, conciseness, general readability, writing style, and native English usage to the best of the editor’s ability.

Best regards,
Wallace Academic Editing

Phone No.: +886-2-2555-5830
Website: http://www.editing.tw
Email: editing@wallace.tw
Address: 3F., No.180, Chang'an W. Rd., Datong Dist., Taipei City
